# ASSESSING STEREOTYPES OF OLDER ADULTS THROUGH DRAWING: IMPLICATIONS FOR PEDAGOGY IN UNDERGRADUATE AGING COURSES

**DOI:** 10.1093/geroni/igac059.2437

**Published:** 2022-12-20

**Authors:** Melannie Pate, Elizabeth Fugate-Whitlock

**Affiliations:** University of North Carolina Wilmington, Wilmington, North Carolina, United States; University of North Carolina Wilmington, Wilmington, North Carolina, United States

## Abstract

Negative stereotypes and ageism toward older adults remain an insidious problem in the United States. These harmful views of older adults can lead to disparities in health care, social isolation, and loss of functional ability. In order to supplement current research on the perceptions of older adults, the researchers focus on the views of undergraduate students before and after taking an aging course. The students included in this study are enrolled in an undergraduate health and aging course or an undergraduate introduction to gerontology course. Before beginning any coursework, students were asked to draw an older adult, using any combination of colored markers available and to write one sentence describing an older adult. At the end of the course, students were again given the same directive to draw an older adult, using any combination of colored markers, and write one sentence describing an older adult. Content analysis was used to analyze the drawings and the phrases. Colors used and depictions of activity in the drawings changed from the first day of class to the end of the course, showing a progression of their views. Drawings were chosen specifically to elicit honest responses and perceptions of older adults. The results of this study can be used to inform pedagogical choices made when teaching courses on aging to undergraduate students.

